# Understanding Human Behavior: Examining the Dark Triad of Personality in the Light of Autism Spectrum Traits

**DOI:** 10.5152/eurasianjmed.2025.25938

**Published:** 2025-09-16

**Authors:** Ezgi Selçuk Özmen, Aslı Enzel Koç

**Affiliations:** 1 Department of Psychiatry, Trabzon Fatih State Hospital, Trabzon, Türkiye; 2 Department of Psychiatry, Sivas Cumhuriyet University, Sivas, Türkiye

**Keywords:** Autism spectrum characteristics, dark triad of personality, human behavior

## Abstract

**Background:**

This study aims to examine the relationship between dark triad personality traits and autism spectrum traits, exploring their impact on social skills, communication, imagination, and empathy. By doing so, it seeks to contribute to psychological assessment and intervention strategies in these areas.

**Methods:**

The study was conducted with a community sample of adults aged 18 and above. Participants were invited through online platforms to complete an anonymous survey. The survey included a sociodemographic form, the short dark triad scale, and the autism spectrum quotient. Inclusion criteria required participants to be at least 18 years old, have sufficient proficiency in Turkish, and not have any known mental or developmental disabilities that could affect their ability to complete the survey. Exclusion criteria included undergoing psychiatric treatment during the study. Prior to participation, informed consent was obtained from all participants, and voluntary participation was emphasized.

**Results:**

The findings revealed a significant negative correlation between autism spectrum traits and Machiavellianism (*r* = −0.247, *P* = .001). This suggests that individuals with communication difficulties tend to struggle with manipulative or strategic social behaviors. Regarding the relationship between autism traits and narcissism, results indicated that higher autism-related imagination scores were associated with lower narcissism levels (*r* = −0.237, *P* = .002). Individuals with fewer autism traits were observed to have a stronger sense of self-worth. This finding suggests that the construction of an exaggerated self-image, a core component of narcissism, may be negatively influenced by deficits in imagination. On the other hand, no significant relationship was found between psychopathy and autism subdimensions. This indicates that the core features of psychopathy, such as emotional detachment and impulsivity, do not directly align with the structured and repetitive behaviors associated with autism.

**Conclusion:**

This study provides important insights into the impact of autism traits and dark triad characteristics on social functioning. Future research should further investigate the cognitive and emotional foundations of these traits using larger and more diverse samples.

**Main Points**

- A significant negative relationship was found between autism spectrum traits and Machiavellianism; communication difficulties are inversely related to manipulative social behaviors.
- Individuals with higher imagination scores showed lower levels of narcissism, suggesting that an exaggerated self-image may be negatively influenced by deficits in imagination.
- No significant relationship was found between psychopathy and autism subdimensions, indicating that traits such as emotional detachment and impulsivity do not align with the structured behaviors typical of autism.
- The findings contribute to psychological assessment and intervention strategies by highlighting how personality traits impact social functioning.
- Overall, while certain autism spectrum traits appear to be meaningfully associated with Machiavellianism and narcissism, the predictive power of these traits on broader dark personality constructs remains limited, underscoring the complexity of their interaction and the need for further research.

## Introduction

The dark triad, conceptualized by Paulhus and Williams (2002),^1^ refers to a cluster of socially aversive personality traits, including narcissism, Machiavellianism, and psychopathy. These traits represent distinct yet interrelated personality dimensions, commonly characterized by tendencies such as egocentrism, emotional detachment, strategic manipulation, and aggression.^2^ Although individuals high in dark triad traits may gain certain advantages in social settings, these tendencies often result in significant negative consequences for interpersonal relationships and group dynamics.^3^ Specifically, Machiavellianism is associated with strategic manipulation and low emotional sensitivity; narcissism is linked to an inflated sense of self-worth and fragile self-esteem; while psychopathy is related to a lack of conscience and marked emotional coldness.^4^^-^^6^

Research exploring dark personality traits has often entertained an “empathic deficit hypothesis,” suggesting that manipulative and exploitative behaviors are rooted in an impaired ability to consider the perspective of others, similar to cognitive patterns observed in autism spectrum conditions.^7^

Although dark triad traits and autistic traits (ATs) emerge from distinct etiological backgrounds, they share notable similarities in their impact on social functioning—particularly in the context of empathy deficits and difficulties in social interactions. Empathy is conceptualized as comprising 2 primary components: cognitive empathy, which involves understanding others’ mental states and is closely linked to theory of mind, and affective empathy, which refers to sharing and responding to the emotions of others.^8^^,^^9^ Impairments in empathic functioning are considered a core feature of dark triad personality traits and also play a significant role in the social-cognitive difficulties observed in neurodevelopmental conditions such as autism spectrum disorder (ASD), which is characterized by deficits in social interaction, communication, and behavioral flexibility.^10^^,^^11^ However, these surface-level resemblances are driven by fundamentally different psychological mechanisms. In ASD, empathic difficulties are primarily rooted in neurodevelopmental limitations, which indirectly impair affective empathy by compromising the perception of nonverbal emotional cues and diminishing attention to socially relevant stimuli.^12^ In contrast, in individuals with dark triad traits, such deficits are typically driven by motivational or strategic factors, often serving manipulative and exploitative purposes.^13^ In the context of narcissism, it is suggested that individuals with this trait employ manipulation strategies that rely on their capacity to comprehend others’ emotions, enabling them to leverage their cognitive empathy skills to influence relationships for their own benefit.^14^ Consequently, while their social behaviors may appear similar, the underlying processes differ markedly. Recognizing these distinctions is essential for accurate diagnosis and the development of targeted intervention strategies.

Another significant similarity is the presence of social motivation deficits, which are observed in both individuals with ATs and those exhibiting dark triad personality characteristics.^15^^,^^16^ Social motivation refers to an individual’s tendency to seek out, engage with, and derive satisfaction from social interactions and relationships. While the manipulative, self-serving, and emotionally detached tendencies of the dark triad reflect a deliberate disinterest in forming genuine social connections, prioritizing self-advancement over mutual relationships, the situation is notably different for autistic individuals.^16^ Although difficulties in forming and maintaining friendships have long been considered core features of autism, often attributed to a lack of motivation and/or cognitive abilities necessary for social interaction, recent research challenges this assumption.^17^^,^^18^ Rather than being unwilling to form relationships, autistic individuals demonstrate different social skills and communication styles, struggle to meet their social needs, and make considerable efforts to adapt, often engaging in camouflaging behaviors to fit in.^18^ While both groups may show reduced social engagement, the motivations and underlying mechanisms differ significantly: dark triad traits reflect a calculated disengagement from social bonds, whereas ATs stem from neurodevelopmental challenges that affect social perception and processing.

Autism spectrum traits are increasingly recognized as dimensional rather than categorical constructs. Recent research suggests that ATs are normally distributed across the general population, indicating that everyone possesses these traits to varying degrees, with pathological expressions typically observed at the extremes.^19^

The primary aim of this study is to examine the relationship between dark triad personality traits (Machiavellianism, narcissism, and psychopathy) and ATs. This study contributes to the literature by clarifying the distinct psychological mechanisms underlying social impairments in dark triad traits and ATs and by identifying potential overlaps and divergences that may inform clinical practice.

Accordingly, the study addresses the following research questions:

1. Is there a significant relationship between dark triad personality traits and ATs?
2. To what extent do the subdimensions of ATs (e.g., social skills, communication, and imagination) predict levels of Machiavellianism, narcissism, and psychopathy?
3. Are there significant differences in dark triad traits between individuals with high and low levels of ATs?

## Material and Methods

### Sample

This study was conducted with a community sample of adults aged 18 years and older. Participants were recruited using non-probability convenience sampling methods through a range of online platforms, including social media (e.g., Instagram, Twitter/X, Facebook), messaging applications (e.g., WhatsApp, Telegram), and email networks. The online survey link, designed and distributed via Google Forms, was disseminated by the researchers through these channels and encouraged further sharing (snowball sampling), potentially amplifying reach among digitally active users. As such, the recruitment strategy primarily targeted individuals who were already engaged in digital environments and comfortable navigating online content.

Upon accessing the survey link, participants were first presented with an informed consent form outlining the purpose of the study, the voluntary nature of participation, and the anonymous handling of all responses. After providing consent, participants were asked to complete a brief sociodemographic questionnaire followed by a set of standardized psychometric instruments. These included self-report measures assessing traits relevant to autism spectrum characteristics (ASCs) and psychopathy.

Inclusion criteria required participants to be at least 18 years old, have sufficient Turkish language proficiency to comprehend and respond to the items, and report no neurological or psychiatric conditions that would significantly impair their ability to complete the survey. Participants who were receiving ongoing psychiatric treatment at the time of the study, including pharmacological or psychotherapeutic interventions, were excluded in order to minimize potential confounding variables related to clinical symptomatology or treatment effects.

Initially, 190 individuals participated in the survey; however, 20 participants who met the exclusion criteria were removed, resulting in a final sample of 170 participants. The survey consisted of 3 components: a sociodemographic questionnaire, the autism spectrum quotient (AQ), and the short dark triad (SD3) scale. Data collection was conducted over a 2-month period between January 2025 and February 2025. The study adhered to the ethical principles outlined in the Declaration of Helsinki, and the research protocol was reviewed and approved by the Ethics Committee of Sivas Cumhuriyet University (Approval No: 2024-11/42; Approval Date: November November 21, 2024). Participants were assured that their responses would remain anonymous and confidential. Before beginning the survey, electronic informed consent was obtained, and participants were required to provide their consent before proceeding.

## Data Collection Instruments

### Sociodemographic Data Form

The sociodemographic data form was designed to collect basic demographic and background information, including age, gender, education level, marital status, and occupation. Additionally, it included questions regarding participants’ psychiatric history, current psychiatric conditions, and legal history, providing a comprehensive profile of the participants.

### Short Dark Triad Scale

The original version of the scale was developed by Jones and Paulhus (2014)^20^ and is referred to as the SD3. The Turkish adaptation of the scale was conducted by Özsoy and colleagues (2017).^21^ The SD3 consists of 3 subscales—Machiavellianism, narcissism, and psychopathy—each comprising 9 items, resulting in a total of 27 items rated on a 5-point Likert scale. Response options range from “1—Strongly Disagree” to “5—Strongly Agree.” The Cronbach’s *α* coefficients reported by Özsoy and colleagues (2017) were 0.70 for Machiavellianism, 0.79 for narcissism, and 0.79 for psychopathy, indicating acceptable internal consistency.

### Autism Spectrum Quotient

The AQ was developed by Baron-Cohen et al^22^ to assess autism spectrum-related traits in adults with typical intelligence. The Turkish adaptation of the AQ was validated by Köse et al (2013),^23^ demonstrating good reliability, with a reported Cronbach’s *α* coefficient of 0.85. The AQ consists of 50 items that evaluate 5 subdomains: social skills, attention switching, attention to detail, communication, and imagination. Each item is rated on a 4-point Likert scale ranging from “definitely agree” to “definitely disagree.” Responses indicative of ATs are scored as 1 point, while other responses are scored as 0, resulting in a total score ranging from 0 to 50. Higher scores reflect a greater presence of autism-related traits, with elevated scores in each subdomain contributing to the overall AQ score.

## Statistical Analysis

The sociodemographic characteristics of the participants were analyzed using descriptive statistical analyses (e.g., frequency, percentage, mean). The relationship between dark triad traits and autism-related traits was examined using Pearson correlation analysis. Differences in dark triad mean scores between participants with and without autism-related traits were analyzed using an independent samples *t*-test. The effectiveness of ASD subdomains in predicting Machiavellianism, narcissism, and psychopathy was assessed using multiple linear regression analysis. A significance level of *P* < .05 was set for all analyses. The normality of the data distribution was evaluated using skewness and kurtosis values (±1.5). All statistical analyses were performed using IBM SPSS 26.0 software (IBM SPSS Corp.; Armonk, NY, USA).

## Results

A total of 170 participants were included in the study. The majority of participants were female (65.3%), while 34.7% were male. Participants were predominantly between the ages of 26-35 (37.6%), followed by 46-65 (22.9%), 36-45 (21.8%), 19-25 (17.1%), and over 65 (0.6%). Regarding education level, 46.5% held a bachelor’s degree, while 27.1% had a master’s/doctorate, 18.2% had a high school diploma, 7.6% an associate’s degree, and 0.6% a middle school degree. Most participants were married or in a civil partnership (61.8%), while 12.9% were in a relationship (not married), 23.5% were single, and 1.8% were divorced, separated, or widowed. In terms of employment status, 67.1% were employed, 14.7% unemployed, and 18.2% students. A total of 37.6% of participants reported a history of psychiatric treatment, and 4.1% reported having legal issues. Detailed demographic information is presented in Table 1.

According to the Pearson correlation analysis, there was a statistically significant negative correlation between Machiavellianism scores and AQ-communication (*r* = −0.247, *P* = .001), AQ-imagination (*r* = −0.165, *P* = .031), and AQ-total (*r* = −0.156, *P* = .042) scores. Similarly, a statistically significant negative correlation was found between narcissism scores and AQ-social skills (*r* = −0.170, *P* = .027), AQ-communication (*r* = −0.176, *P* = .022), AQ-imagination (*r* = −0.237, *P* = .002), and AQ-total (*r* = −0.183, *P* = .017) scores. This information is detailed in Table 2.

In the hierarchical linear regression analysis, Machiavellianism scores were used as the dependent variable, while AQ subscale scores served as the independent variables. The model was constructed to assess the impact of AQ subscale scores on Machiavellianism. The analysis revealed that AQ subscale scores accounted for 9% of the variance in Machiavellianism scores in a statistically significant manner (*F* = 3.31, *P* = .007). A more detailed examination of the model showed that only the AQ-communication subscale scores were significant predictors of Machiavellianism scores (*P* = .001, CI: −1.577 to −0.387). These findings are presented in detail in Table 3.

Similarly, hierarchical linear regression analysis indicated that 8% of the variance in narcissism scores was significantly explained by AQ subscale scores (*F* = 2.98, *P* = .013). Further analysis showed that only the AQ-imagination subscale (*P* = .018, CI: −1.047 to −0.099) was a statistically significant predictor of narcissism. These findings are detailed in Table 4.

In contrast, hierarchical linear regression analysis revealed no statistically significant effect of AQ subscale scores on psychopathy scores ( > .05), as shown in Table 5.

This regression analysis examined the effects of AQ subdimensions (social skills, attention switching, attention to detail, communication, and imagination) on total dark triad scores. However, the results indicated that none of the variables significantly predicted dark triad traits. The *P* values for all subdimensions ranged from 0.143 to 0.970, exceeding the conventional threshold of 0.05 for statistical significance. These findings suggest that ASCs do not have a meaningful impact on dark triad personality traits and that the explanatory power of the model is notably low, as shown in Table 6.

According to independent samples *t*-test results, participants with AQ scores below 22 (AQ < 22) had significantly higher mean Machiavellianism scores (*t* = 3.51, *P* = .001) and higher mean narcissism scores (*t* = 3.66, *P* < .001) compared to those with AQ scores of 22 or above (AQ ≥ 22). This comparison is illustrated in Table 7.

## Discussion

This study investigated the associations between ASCs and the dark triad personality traits—Machiavellianism, Narcissism, and Psychopathy. The findings reveal a nuanced and important distinction: while specific ASC subdimensions, particularly communication difficulties and limitations in imagination, were negatively associated with Machiavellian and narcissistic traits, no significant relationship was found between psychopathy and any ASC subdomain. This suggests that individuals with higher ASC traits may demonstrate fewer manipulative and self-centered behaviors typically associated with dark personality profiles. The core features of ASC—such as reduced mentalizing capacity, challenges in perspective-taking, and a tendency toward concrete, rule-based thinking—may limit the development of complex social strategies like manipulation and self-promotion, which are central to Machiavellianism and narcissism. In contrast, psychopathy—characterized by emotional coldness, impulsivity, and a lack of empathy—appears to operate through fundamentally different psychological mechanisms. Moreover, the low predictive power of ASC traits on overall dark triad scores highlights the need for careful interpretation of aggregate measures, as combining distinct personality traits into a single composite may obscure meaningful associations.

### Relationship Between Machiavellianism and Autism Spectrum Characteristics

The findings revealed a clear negative association between communication difficulties and Machiavellianism. Individuals with higher levels of communication challenges—one of the core features of ASC—tended to report lower Machiavellian traits. This suggests that the social-cognitive limitations inherent in ASC, such as reduced theory of mind abilities, difficulties in perspective-taking, and challenges in understanding deception, may limit the ability to engage in manipulative or strategic social behaviors. Machiavellianism typically involves a calculated, goal-oriented use of social influence, requiring a degree of mentalizing and empathy skills that may be impaired in individuals with pronounced ATs.^7^^,^^24^

Interestingly, participants with lower ASC scores (AQ < 22) exhibited higher levels of Machiavellianism. This pattern implies that individuals with fewer autism-related difficulties may possess a greater capacity to navigate complex social dynamics, adapt their behavior to social contexts, and employ manipulative strategies when necessary. In contrast, those with stronger ASC profiles may lack the flexibility and social intuition required for such behaviors, resulting in lower levels of Machiavellian tendencies.^7^ While Machiavellianism is associated with lower empathy scores, this does not necessarily indicate an inability to understand others’ emotions; rather, it may reflect a reduced willingness to show compassion.^25^^,^^26^ Empathy measures often capture a tendency to engage in compassionate behavior, not the actual ability to understand others’ emotions.^27^ Therefore, lower Machiavellianism scores in individuals with greater communication difficulties may reflect limited social-cognitive skills necessary for manipulative behavior.

### Relationship Between Narcissism and Autism Spectrum Characteristics

Another important finding of the study concerned the relationship between narcissism and ASC. Specifically, individuals with low imagination scores exhibited higher levels of narcissism. It was found that limitations in imagination may hinder the development of narcissistic traits, which are often characterized by exaggerated self-images and self-aggrandizing narratives that require imaginative and creative thinking. Among autistic individuals, such limitations in imaginative capacity may restrict the development of narcissistic tendencies.^17^

Individuals with lower ASC scores (AQ < 22) were observed to have higher narcissism scores. This suggests that individuals with fewer autistic characteristics may be more inclined to perceive themselves as important and valuable—possibly as a social adaptation strategy for self-promotion. The core narcissistic features of “grandiose fantasy” and excessive imagination play a critical role in reinforcing such self-enhancing beliefs.^28^^,^^29^

### Relationship Between Psychopathy and Autism Spectrum Characteristics

Interestingly, no significant relationship was found between psychopathy and ASC in this study. The core components of psychopathy—emotional coldness and impulsivity—do not directly overlap with ASD. This finding suggests that while both psychopathy and ASC involve deficits in empathy, the nature of these deficits differs. In psychopathy, the lack of empathy is emotionally detached and calculative, whereas in autism, empathy difficulties arise from cognitive challenges in social interaction.^30^

This study contradicts perspectives in the literature suggesting that the empathy deficits in psychopathy might be confused with the social-cognitive challenges seen in autism. Instead, it shows that the similarities between psychopathy and autism are superficial and that these conditions are rooted in fundamentally different cognitive and emotional mechanisms.

### Relationship Between Total Dark Triad and Autism Spectrum Characteristics

The current findings indicate that AQ subscale scores did not significantly predict either total dark triad scores or psychopathy, with both models accounting for only 3% of the variance and failing to reach statistical significance. This contrasts with the models predicting Machiavellianism and narcissism, wherein specific AQ dimensions—particularly communication and imagination—emerged as significant predictors. The correlation matrix further supports this distinction, showing that psychopathy was not significantly correlated with any AQ subdimension or the total AQ score, whereas Machiavellianism and narcissism demonstrated consistent negative correlations with key AQ domains. These findings suggest that psychopathy may represent a distinct personality construct with limited overlap with ATs. Consequently, the inclusion of psychopathy in the total dark triad composite may obscure meaningful associations that are primarily driven by Machiavellian and narcissistic tendencies. A plausible explanation is that aggregating conceptually and behaviorally distinct traits into a single score may mask trait-specific patterns by averaging out unique variance, thereby limiting the interpretability of psychological correlates. These results underscore the importance of examining dark triad traits individually, particularly when investigating their relationship with neurodevelopmental features such as ATs.

Several methodological limitations should be considered when interpreting the findings of this study. The online distribution of the survey via social media platforms restricted participation to individuals with internet access and active social media use, which may have limited the representativeness of the sample. Since participation was voluntary, the study may have attracted individuals with a particular interest in or knowledge of the topic, potentially introducing self-selection bias. Moreover, as the data were collected through self-report measures, responses may have been influenced by social desirability bias, wherein participants might present themselves in a more socially acceptable manner. The inability of researchers to verify the accuracy of these responses introduces concerns regarding the reliability of the data. Individuals lacking internet access or possessing limited digital literacy were likely underrepresented in the sample, thereby reducing its overall diversity. Additionally, the questionnaire was administered solely in Turkish, excluding individuals without sufficient Turkish language proficiency and thus limiting the linguistic and cultural inclusiveness of the study.

It is also noteworthy that the instruments used—namely the AQ and the SD3—are screening tools rather than diagnostic measures. This raises questions regarding the sensitivity and comprehensiveness of the instruments. Specifically, these tools may primarily capture a limited subset of the autism spectrum, most likely high-functioning individuals, which calls into question the generalizability of the findings across the full spectrum. The use of well-validated scales in this study provides a solid quantitative foundation for the findings, though further research with more comprehensive tools is encouraged. Additionally, while this study does not directly address the neurobiological underpinnings of psychopathy and autism spectrum traits, its findings may inform future research efforts, including neuroimaging and neuropsychological assessments, to better understand the distinct mechanisms underlying these constructs.

In conclusion, this study highlights the distinct nature of ASCs and dark triad personality traits. While communication difficulties and limitations in imagination were associated with lower levels of Machiavellianism and narcissism, no significant link was found between psychopathy and ATs. These findings suggest that the cognitive and social challenges in autism differ fundamentally from the interpersonal strategies and emotional detachment characteristic of dark triad traits. Future studies should explore these relationships in greater depth, using diverse methodologies and samples to clarify the complex interplay between neurodevelopmental features and dark personality traits.

## Figures and Tables

**Table 1.** Demographic Characteristics of Participants

Aa		n	%	Ort
Age (years)	19-25	29	17.1	22
	26-35	64	37.6	
	36-45	37	21.8	
	46-65	39	22.9	
	Over 65	1	0.6	
Gender	Male	59	34.7	
	Female	111	65.3	
Education level	Bachelor’s degree	79	46.5	
	High school	31	18.2	
	Middle school	1	0.6	
	Associate’s degree	13	7.6	
	Master’s/doctorate	46	27.1	
Relationship status	Married/civil partnership	105	61.8	
	In a relationship (not married)	22	12.9	
	Single (no relationship)	40	23.5	
	Divorced/separated/widowed	3	1.8	
Employment status	Employed	114	67.1	
	Unemployed	25	14.7	
	Student	31	18.2	
Psychiatric history	Previous treatment (yes)	64	37.6	
Legal issues	Yes	7	4.1	
	No	163	95.9	

**Table 2.** Correlation Between the Dark Triad and Autism Spectrum Traits

		1	2	3	4	5	6	7	8
1- Machiavellianism	*r*	–							
	*P*	–							
2- Narcissism	*r*	0.357							
	*P*	<.001							
3- Psychopathy	*r*	0.294	0.277						
	*P*	<.001	<.001						
4- AQ–social skills	*r*	−0.077	**−0.170**	0.015					
	*P*	.321	**.027**	.847					
5- AQ–attention shift	*r*	0.037	0.023	0.131	0.288				
	*P*	.635	.768	.087	<.001				
6- AQ–attention detail	*r*	0.021	0.047	0.047	−0.194	−0.142			
	*P*	.783	.540	.541	.011	.065			
7- AQ–communication	*r*	**−0.247**	**−0.176**	0.019	0.487	0.329	−0.069		
	*P*	**.001**	**.022**	.803	<.001	<.001	.371		
8- AQ–imagination	*r*	**−0.165**	**−0.237**	−0.040	0.344	0.154	−0.097	0.290	
	*P*	**.031**	**.002**	.602	<.001	.044	.209	<.001	
9- AQ–total	*r*	**−0.156**	**−0.183**	0.061	0.717	0.565	0.182	0.752	0.567
	*P*	**.042**	**.017**	.426	<.001	<.001	.018	<.001	<.001

*r* values represent Pearson correlation coefficients. *P* values indicate the statistical significance of the relationships.

Bold values indicate statistically significant correlations at p < 0.05.

**Table 3.** The Effect of AQ Subscales on Explaining Machiavellianism Scores

	Unstandardized Coefficients		Standardized Coefficients Beta	t	*P*	95.0% CI	
	B	SE				LL	UL
Constant	27.229	2.498		10.901	<.001	22.297	32.161
AQ–social skills	0.238	0.294	0.073	0.811	.419	−0.342	0.818
AQ–attention shift	0.534	0.322	0.133	1.658	.099	−0.102	1.170
AQ–attention detail	0.081	0.277	0.022	0.292	.771	−0.466	0.628
AQ–communication	−0.982	0.301	**−0.288**	−3.259	.001	−1.577	−.387
AQ–imagination	−0.546	0.351	−0.125	−1.559	.121	−1.239	0.146

*R*^2^ = 0.09; *F* = 3.31; *P* = .007. Multivariate linear regression analysis.

LL, lower limit; SE, standard error; UL, upper limit.

Note: In the regression analysis, the AQ–Communication variable showed a significant negative relationship (β = -0.288, t = -3.259, p = 0.001). The effects of the other variables were not statistically significant (p > 0.05). The confidence intervals also support the reliability of this relationship.

**Table 4.** The Effectiveness of AQ Subscales in Explaining Narcissism Scores

	Unstandardized Coefficients		Standardized Coefficients Beta	*t*	*P*	95.0% CI	
	B	SE				LL	UL
Constant	27.214	1.710		15.914	<.001	23.838	30.591
AQ–social skills	−0.165	−0.201	−0.074	−0.819	.414	−0.562	0.232
AQ–attention shift	0.320	0.220	0.117	1.450	.149	−0.116	0.755
AQ–attention detail	0.056	0.190	0.023	0.295	.769	−0.319	0.430
AQ–communication	−0.281	0.206	−0.121	−1.362	.175	−0.688	0.126
AQ–imagination	−0.573	0.240	−0.192	−2.387	**.018**	−1.047	−0.099

*R*^2^ = 0.08; *F* = 2.98; *P* = .013. Multivariate linear regression analysis.

LL, lower limit; SE, standard error; UL, upper limit.

**Table 5.** The Effectiveness of AQ Subscales in Explaining Psychopathy Scores

	Unstandardized Coefficients		Standardized Coefficients Beta	*t*	*P*	95.0% CI	
	B	SE				LL	UL
Constant	17.169	1.815		9.462	<.001	13.586	20.752
AQ–social skills	0.024	0.213	0.010	0.112	.911	−0.397	0.445
AQ–attention shift	0.426	0.234	0.151	1.819	.071	−0.036	0.888
AQ–attention detail	0.163	0.201	0.064	0.812	.418	−0.234	0.561
AQ–communication	−0.035	0.219	−0.015	−0.160	.873	−0.467	0.397
AQ–imagination	−0.174	0.255	−0.057	−0.683	.496	−0.677	0.329

*R*^2^ = 0.03; *F* = 0.84; *P* = .521. Multivariate linear regression analysis.

LL, lower limit; SE, standard error; UL, upper limit.

**Table 6.** The Effectiveness of AQ Subscales in Explaining Total Dark Triad Scores

	Unstandardized Coefficients		Standardized Coefficients Beta	*t*	*P*	95.0% CI	
	B	SE				LL	UL
Constant	70.143	6.939		10.108	<.001	56.364	83.923
AQ–social skills	−0.945	0.640	−0.159	−1.477	.143	−0.397	0.445
AQ–attention shift	0.873	0.766	0.124	1.140	.257	−0.036	0.888
AQ–attention detail	−0.379	0.693	−0.056	−0.547	.585	−0.234	0.561
AQ–communication	0.356	0.678	0.056	0.525	.600	−0.467	0.397
AQ–imagination	0.034	0.909	0.004	0.037	.970	−0.677	0.329

*R*^2^ = 0.03; *F* = 0.66; *F* = 0.66; *P* = .658. Multivariate linear regression analysis.

LL, lower limit; SE, standard error; UL, upper limit.

**Table 7.** Comparison of Dark Triad Traits Between Participants with and Without Autistic Traits

	AQ (<22) (n = 71)		AQ (≥22) (n = 99)		t	*P*
	Mean	SD	Mean	SD		
Machiavellianism	27.08	5.99	23.32	7.97	3.51	.001
Narcissism	26.51	4.70	23.85	4.65	3.66	<.001
Psychopathy	19.35	5.16	19.31	4.76	0.06	.955

*P* values calculated using independent samples *t*-test.

SD, standard deviation.

Note: Significant differences were found between the groups with AQ scores <22 and ≥22 in Machiavellianism and Narcissism scores (t = 3.51, p = 0.001; t = 3.66, p < 0.001, respectively). However, no significant difference was observed in Psychopathy scores between the groups (t = 0.06, p = 0.95).

## Data Availability

The data that support the findings of this study are available on request from the corresponding author.
